# Reduced durability of hybrid immunity to SARS-CoV-2 in immunocompromised children

**DOI:** 10.3389/fimmu.2024.1502598

**Published:** 2024-12-17

**Authors:** Youjia Zhong, Amuthavalli Kottaiswamy, Chen Xiang Ang, Hui’ En Li, Gaik Chin Yap, Carina J. X. Tay, Nurul Elyana Osman, Siti Namirah Binte Roslan, Chee Wah Tan, Wee Chee Yap, Elizabeth Y. Ang, Pauline P. L. Chan Ng, Hui Kim Yap, Liangjian Lu, Marion M. Aw, Sivaraman V. Karthik, Seng Hock Quak, Thuan Chong Quah, Elizabeth H. Tham, Lynette P. Shek, Eng Eong Ooi

**Affiliations:** ^1^ Department of Pediatrics, Yong Loo Lin School of Medicine, National University of Singapore (NUS), Singapore, Singapore; ^2^ Program of Emerging Infectious Diseases, Duke-NUS Medical School, Singapore, Singapore; ^3^ Khoo Teck Puat-National University Children’s Medical Institute, National University Health System, Singapore, Singapore; ^4^ Infectious Diseases Translational Research Program, Department of Microbiology and Immunology, Yong Loo Lin School of Medicine, NUS, Singapore, Singapore; ^5^ Viral Research and Experimental Medicine Center, SingHealth Duke-NUS Academic Medical Center, Singapore, Singapore; ^6^ Department of Clinical Translational Research, Singapore General Hospital, Singapore, Singapore

**Keywords:** adaptive immunity, COVID-19, memory B cells, T cells, vaccine immunogenicity, correlates of protection, vaccine durability

## Abstract

**Background:**

In endemic COVID-19, immunocompromised children are vulnerable until vaccinated but the optimal primary vaccination regime and need for booster doses remains uncertain.

**Methods:**

We recruited 19 immunocompromised children (post-solid organ transplantation, have autoimmune disease or were on current or recent chemotherapy for acute lymphoblastic leukemia), and followed them from the start of primary vaccination with BNT162b2 mRNA SARS-CoV-2 until 1-year post-vaccination. We investigated the quality of vaccine immunogenicity, and longevity of hybrid immunity, in comparison to healthy children.

**Results:**

Immunocompromised children failed to produce T cell and memory B cell (MBC) responses reaching thresholds of protection after 2 doses; a third dose however improved both responses. Initially robust hybrid immunity demonstrated significantly more decline in T cell and MBC responses in immunocompromised compared to healthy children, to levels below the protective threshold by month 12.

**Discussion:**

Immunocompromised children may benefit from a 3-dose primary vaccination regime, with yearly or twice-yearly booster doses for sustained immunity.

## 1 Introduction

COVID-19 has, with widespread partial immunity from vaccination and infection, transitioned from a pandemic problem to global endemicity, joining other respiratory viruses as one of the causes of annual and cyclical epidemics. As new variants of concern continue to emerge, COVID-19 vaccination will likely continue to feature prominently in public health programs worldwide, targeting those vulnerable to severe COVID-19. One such group is immunocompromised children who remain completely vulnerable to COVID-19 until they are vaccinated. However, as both vaccine immunogenicity and quality of hybrid immunity have not been sufficiently studied in this population, the optimal primary vaccination regime and need for booster doses is still uncertain.

We recently defined the correlates of protection against symptomatic SARS-CoV-2 infection (1). Prior to hybrid immunity, whereupon neutralizing antibodies reach titers sufficient for protection, 2-dose vaccine-induced T and memory B cell (MBC) responses correlated with durable protection and with strong predictive power for preventing symptomatic infection (1).

In this study, we compared vaccine immunogenicity and hybrid immunity between immunocompromised and healthy children, using the previously defined correlates of protection to infer the minimally-required vaccination regime to protect immunocompromised children from symptomatic SARS-CoV-2 infection.

## 2 Materials and methods

### 2.1 Experimental model and subject details

We conducted a prospective cohort study “MARkers of Vaccine Efficacy and Longevity in SARS-CoV-2 (MARVELS)” in healthy and immunocompromised children aged 5 to 12 years old. The study protocol was approved by the National Healthcare Group Domain Specific Review Board (NHG DSRB) (2021/00945, 2021/00984, 2021/01040). Children were recruited from the general population, through advertisements around the National University Hospital, Singapore and in the community with written parental consent and written subject assent. All children had no history of SARS-CoV-2 infection prior to enrollment, and were negative for anti-nucleocapsid antibodies. Demographic characteristics including gender are parent- or self-reported, and not considered for enrollment. Decision to receive the vaccination was based on parental discretion, and healthy children were inoculated with two doses of 10mcg monovalent BNT162b2 at days 0 and 21. Immunocompromised children all received two 10mcg doses of monovalent BNT161b2 at days 0 and 21, and the inclusion of a third dose of 10mcg monovalent BNT162b2 as part of the primary vaccination regime, 2 months after dose 2, was based on discretion of the primary physician looking after the child. The interval between the first and second dose was extended to 3 months if the child acquired natural SARS-CoV-2 infection after dose 1. Among children who completed their primary vaccination regime, several received a booster dose at 5 months or later after the last dose, also at parental discretion for healthy children, and physician discretion for immunocompromised children. Healthy children were recruited between 20 December 2021 and 8 March 2022, while immunocompromised children were recruited between 19 December 2021 and 1 April 2022.

All children underwent venipuncture 10 days after dose 1, and at 3, 6 and 12 months after the last dose of primary vaccination. In addition, immunocompromised children who received 3 doses for primary vaccination also underwent venipuncture 6 weeks after dose 2.

### 2.2 Total immunosuppression score

Patients were scored for total immunosuppression using a total immunosuppression score developed by the authors and largely based on literature (2, 3). This score is calculated by first assigning each immunosuppressive agent a score per dose by weight (mg/m^2^/day), and then summing together the scores for all immunosuppressive agents that each subject was on at recruitment (**Supplementary Table 2**).

### 2.3 Identification of antigen rapid test-positive symptomatic SARS-CoV-2 infections

Parents were trained to administer a SARS-CoV-2 ART testing at home if the child developed symptoms suggestive of COVID-19 anytime during the follow up period, according to prevailing national guidelines for active surveillance and community treatment during the COVID-19 pandemic in Singapore. ART kits were certified by the Health Sciences Authority of Singapore and freely distributed by the Ministry of Health. ART+ symptomatic SARS-CoV-2 infections were reported to the study team within 72 hours, and a symptom diary was filled up by the study team based on a phone interview conducted within 72 hours.

### 2.4 Serological analysis

#### 2.4.1 Anti-spike IgG and anti-nucleocapsid antibodies

Anti-S IgG was quantified using enzyme-linked immunosorbent assay (ELISA), as previously described (1, 4). Briefly, two high-binding 96-well ELISA microplate (Greiner) were coated with 1ug/mL Wuhan-Hu-1 S hexapro protein diluted in PBS and incubated at room temperature (RT) for 45 minutes. Plates were washed with PBS-T (0.05% Tween-20) and incubated with blocking buffer (PBS with 3% BSA) at RT for 1 hour (5). Plasma was diluted 200x and 5000x in blocking buffer, while a standard antibody (anti-SARS-CoV-2 S RBD Neutralizing Antibody, Acrobiosystems) was serially diluted 10x for the standard curve. Blocked plates were washed and then incubated with diluted plasma and antibody standard in duplicates at RT for 1 hour. Plates were washed and then incubated with HRP-IgG secondary antibody (Life Technologies) at 10,000x dilution at RT for 1h. Lastly, plates were washed and then detection reagent 3,30,5,50-Tetramethylbenzidine (TMB) (Thermo fisher) was added. Reaction was quenched with 1M Sulfuric acid/phosphoric acid. Sample optical density (OD) was measured with a spectrophotometer at 450nm, and concentrations in U/ml were interpolated from the standard curve using GraphPad Prism 10.0.2.

Anti-N antibodies were detected using Elecsys^®^ Anti-SARS-CoV-2 immunoassay (Roche) for qualitative detection of total antibodies against N antigen, which uses a sandwich ELISA against recombinant N. Manufacturer’s instructions were followed.

#### 2.4.2 Surrogate virus neutralization assay (Wuhan-Hu-1 only)

Neutralizing antibodies against Wuhan-Hu-1 S were measured, according to manufacturer’s instructions, using a commercial surrogate virus neutralization assay cPASS (GenScript) which is based on an ELISA measuring binding of Receptor-Binding Domain (RBD) to human angiotensin-converting enzyme 2 (ACE2) (1). This assay probes for antibodies inhibiting recombinant SARS-CoV-2 S protein binding to the hACE2 receptor. Technical duplicates were used for this assay. Percentage inhibition of RBD-hACE2 binding was computed using the following equation: % inhibition = (1 – [(OD of serum + RBD)/(OD of negative control + RBD)]) x 100. As described by the cPASS kit, a cut-off of 20% was used as the lower limit of positivity. Samples were serially diluted until % inhibition was below 50%, and half-maximal inhibitory concentration was interpolated using GraphPad Prism 10.0.2.

#### 2.4.3 Pseudotyped virus neutralization assay (for Wuhan-Hu-1 and SARS-CoV-2 variants)

Neutralizing antibodies against VOCs were measured with a pseudotyped virus neutralization assay (pVNT), as previously described (1) Human lung carcinoma epithelial (A549, ATCC CRM CCL-185) cells were grown and maintained in RPMI-1640 supplemented with 10% FBS. Human ACE2 gene in pFUGW vector was introduced into A549 cells by lentivirus transduction1 and maintained in RPMI 1640 supplemented with 10% FBS and 15 µg/ml of blasticidin. Human embryo kidney (HEK293T, ATCC CRL-3216) cells were grown and maintained in DMEM supplemented with 10% FBS. SARS-CoV-2 parental (Wuhan-Hu-1), Beta, Delta, Omicron BA.2, Omicron XBB.1.16 (E180V, T478R) and EG.5.1 (F456L, Q52H) full-length spike pseudotyped viruses were produced by transfecting 20 µg of pCAGGS spike plasmid into 5 million HEK293T cells using FuGENE 6 (Promega) (6). At 24 h post-transfection, the transfected cells were infected with VSVΔG luc seed virus at MOI of 5 for 2h. After two PBS washes, infected cells were replenished with DMEM 10% FBS supplemented with 1:5000 diluted anti-VSV-G mAb (Clone 8G5F11, Kerafast). Upon 80% cytopathic effect, pseudotyped viruses were harvested by centrifugation at 2,000 x g for 5 min. Pseudoviruses (~ 3 million RLU) were pre-incubated with four-fold serial diluted test serum in a final volume of 50 ul for 1h at 37°C, followed by infection of A549-ACE2 cells. At 20-24 h post-infection, an equal volume of ONE-Glo luciferase substrate (Promega) was added and the luminescence signal was measured using the citation 5 microplate reader (BioTek) with Gen5 software version 3.10. The 50% neutralizing titer (NT50) was interpolated using GraphPad Prism 10.0.2.

### 2.5 Cytokine release assay for S- and N-reactive T cell responses

T cell responses were quantified with a cytokine release assay, a validated method of quantifying T cell responses that has good correlation to T cell ELISPOT (1, 7). Fresh peripheral blood was stimulated with 55 overlapping 15-mer peptide pools covering the immunogenic regions of the Wuhan-Hu-1 SARS-CoV-2 S protein (representing 40.5% of the whole S protein) (GenScript) before and after vaccination. Similar overlapping peptide pool of SARS-CoV-2 N protein (spanning the entire N protein) was also used to stimulate peripheral blood to test for prior SARS-CoV-2 infection. Freshly drawn whole blood was mixed with RPMI and stimulated with the indicated N or S peptide pool at 2ug/ml, or with 1.25% DMSO as a control. After 16 hours of incubation, the supernatant (plasma) was collected and stored at -80°C until analysis. Cytokine concentrations in the plasma was quantified using an Ella machine (ProteinSimple) with microfluidic multiplex cartridges that measured Th1 specific cytokines interferon-γ (IFN-γ) and interleukin-2 (IL-2) for both adults and children. In addition, Th2 specific cytokines interleukin-4, interleukin-5 and interleukin-13, and other cytokines tumor necrosis factor-α, Granzyme-B and interleukin-10 were quantified for children, according to the manufacturer’s instructions (ProteinSimple). The levels of cytokines present in the plasma of DMSO controls were subtracted from the corresponding N or S stimulated samples. Technical duplicates were used for this assay.

### 2.6 Isolation of peripheral blood mononuclear cells

Peripheral blood was collected from all individuals in heparin-containing tubes, and PBMCs from all collected blood samples were isolated by Ficoll-Paque density gradient centrifugation. PBMCs were cryopreserved in liquid nitrogen until analysis.

### 2.7 Spike specific memory B cell quantification, culture and ELISPOT

S+ MBCs were quantified using flow cytometry as previously described (1). Briefly, thawed PBMCs were first enriched for B cells using pan B cell isolation kit (Miltenyi, Germany), according to manufacturer’s guidelines. Biotinylated full-length Wuhan-Hu-1 S proteins (Miltenyi, Germany) were incubated with fluorescently labeled streptavidin (SA) for 15 minutes at room temperature (1). Cells were stained with an antibody cocktail containing CD3, CD19, CD21, CD27, CD38, CD138, CD71, IgA, IgG, IgD, IgM and 7-AAD for 30minutes at 4°C prior to acquisition on the LSR Fortessa flow cytometer (BD). S+ MBC were defined as live CD3-CD19+IgD-CD27+CD38-/+S bispecific cells, while S+ PB were defined as live CD3-CD19+IgD-CD27+CD38++/S bispecific cells.

### 2.8 Identification of asymptomatic SARS-CoV-2 infections

N-specific antibodies, anti-S IgG, as well as N-reactive T cell responses, were used to identify subjects who were asymptomatically infected. N-reactive T cell responses were selected as a large proportion of SARS-CoV-2-convalescent individuals develop T cell responses against N, and its absence among pre-pandemic donor samples demonstrates its low cross-reactivity with seasonal coronaviruses (8). At each time point, any 1) newly positive anti-N antibodies, 2) 4-fold rise in anti-S IgG in the absence of vaccination, or 3) significant increase in IFN-γ or interleukin-2, were taken to represent an interim asymptomatic SARS-CoV-2 infection. Significant increase in IFN-γ or interleukin-2 was defined as either 1) 10-fold increase of cytokine level from baseline, 2) 10-fold increase of cytokine level from the last visit, or 3) cytokine level 10 times above the threshold of positivity. As there is non-specific T cell activation up to 4 weeks post vaccination, N-reactive T cell responses at Day 10 post dose 1 were not used for identification of asymptomatically infected individuals (7). As N-reactive T cell responses were not used as an exclusion criterion for enrolment in this study, children with asymptomatic SARS-CoV-2 infections as evidenced by high N-reactive T cell responses as baseline were included in the study; these were excluded when studying vaccine-only immunity but included when studying hybrid immunity.

### 2.9 Quantification and statistical analysis

Clinical data was collected in REDCap version 14.2.2, and exported for analysis into Microsoft Excel. Python 3.9.2 was used to combined immunological parameters and clinical data. Statistical analyses were conducted using GraphPad Prism 10.0.2 and IBM SPSS 29. Two-tailed Mann-Whitney U test was used for comparison of unpaired continuous data between two groups. Spearman correlation was used to analyze the association between non-parametric continuous variables. Logrank (Mantel-Cox) test was used to compare groups for Kaplan-Meier survival curves. p<0.05 level of confidence was accepted for statistical significance. Figures were created using GraphPad Prism 10.0.2 and BioRender. All box and whiskers plots show median (center line), interquartile range (box) and range (whiskers).

## 3 Results

### 3.1 Participant characteristics

**Figure 1:**
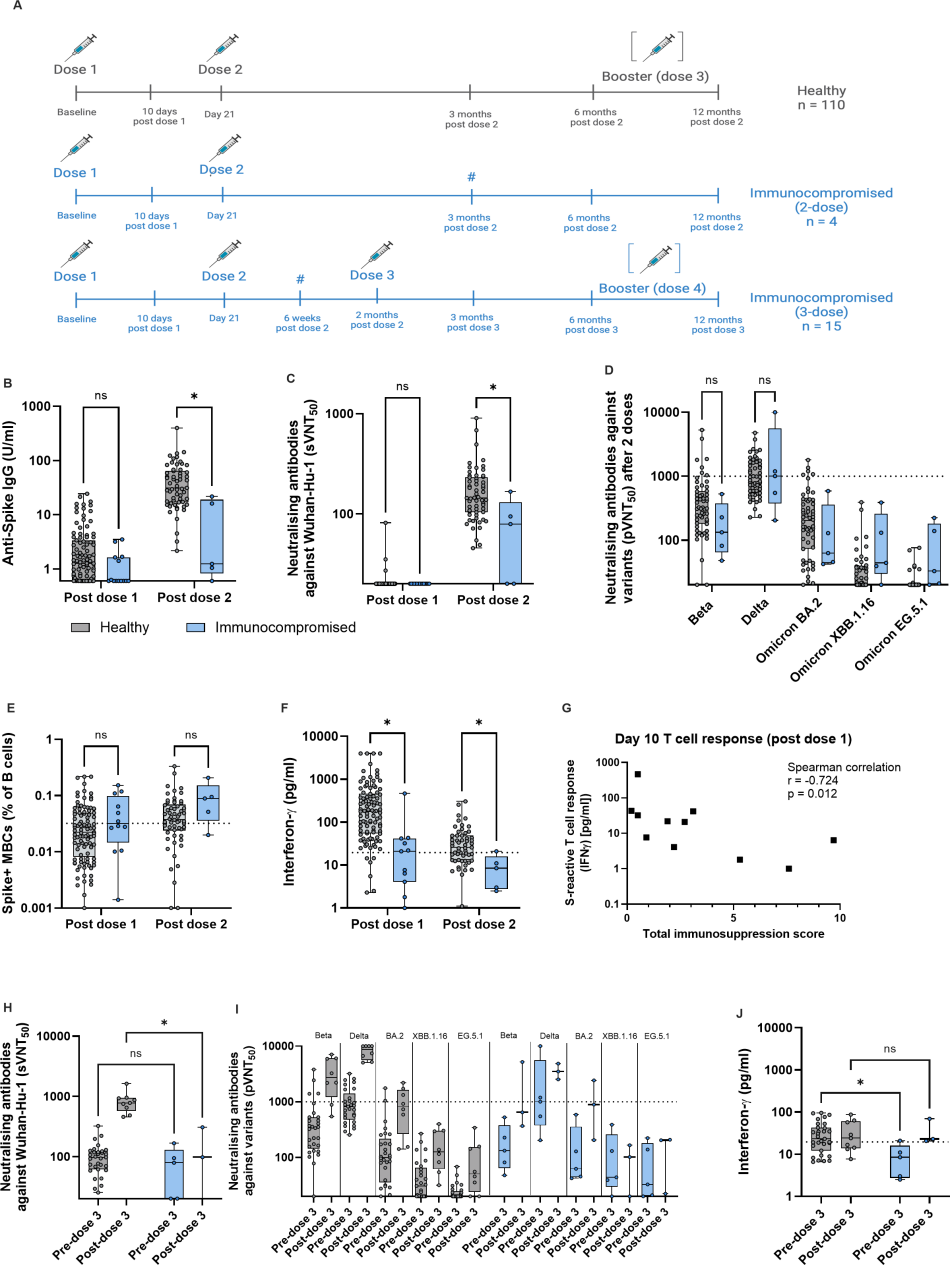
Immunocompromised children have reduced immunogenicity to two vaccine doses, but this is improved upon after dose 3. **(A)** Schematic of study schedule for healthy (grey) and immunocompromised (blue) children, all aged 5-12 years, in the MARkers of Vaccine Efficacy and Longevity in SARS-CoV-2 (MARVELS) study up to 12 months post vaccination. Healthy children (n = 116) were given two doses of monovalent 10mcg BNT162b2 on day 0 and 21 of the study. Immunocompromised children (n = 19) were given the same 2 doses, and a third dose of 10mcg BNT162b2 2 months after dose 2 as part of their primary vaccination regime, according to physician discretion. Venous blood was drawn at day 10 after dose one, 6 weeks post dose 2 only for immunocompromised children who received 3 doses of vaccine, and 3, 6 and 12 months after the last dose of primary vaccination. Children who acquired natural SARS-CoV-2 infection (both symptomatic and asymptomatic) or received a booster dose before each sampling time point were excluded from comparison for that time point. Thus, for the post dose 1 time point, n = 110 healthy and n = 16 immunocompromised, for the post dose 2 time point, n = 60 healthy and n = 7 immunocompromised, and for the post dose 3 time point, n = 3 immunocompromised. For the post-dose 2 and pre-dose 3 time points for immunocompromised children, data from 4 children who received a 2-dose primary vaccination regime and 15 children who received a 3-dose regime were combined (marked with #). **(B)** Anti-Spike (S) IgG titers post dose 1 and post dose 2 of vaccine. **(C)** Titers of antibodies that neutralized 50% of Wuhan-Hu-1 S protein RBD binding to ACE2, as measured using surrogate virus neutralization test (sVNT_50_). **(D)** Titers of antibodies that neutralized 50% of indicated variants, as measured using pseudovirus neutralization test (pVNT_50_), for post dose 2 time point. **(E)** Percentage of S+ memory B cells (MBCs) out of total B cells. **(F)** S-reactive T cell responses measured by post-stimulation interferon (IFN)-γ. **(G)** Correlation of total immunosuppression score with S-reactive T cell responses as measured by post-stimulation interferon-γ 10 days after dose 1 of vaccine. **(H)** pVNT_50_ before and after dose 3 for healthy and immunocompromised children. **(I)** Titers of antibodies that neutralized 50% of indicated variants, as measured using pseudovirus neutralization test (pVNT_50_), for before and after dose 3. **(J)** S-reactive T cell responses before and after dose 3. For all box-and-whisker graphs, the top and bottom boundaries of the boxes indicate upper and lower quartiles, respectively, the center line indicates median and the whiskers represent the range. For all panels, a two-tailed Mann-Whitney U test was used for comparisons between groups. Ns, not significant, *P ≤ 0.05. The schematic was created in BioRender.com.

Immunocompromised children (n = 19) aged 5-12 years were recruited between 19 December 2021 and 1 April 2022 and followed up until 1 year after the end of the primary vaccination regime (**Figure 1A**). These children had neither clinical history, nor serological evidence of prior SARS-CoV-2 infection. Immunocompromised children were either post-solid organ transplantation, had autoimmune disease or were on current or recent (completed less than 3 months ago) chemotherapy for acute lymphoblastic leukemia (**Supplementary Table 1**). Most of the immunosuppressive medications were T cell immunosuppressants, with total immunosuppression score ranging from 0.2 to 9.7. The healthy children cohort (n = 116) were of similar ages and enrolled during the same period as the immunocompromised children, as previously described (**Figure 1A**) (1).

All healthy children received 2 doses of monovalent 10 mcg BNT162b2 21 days apart. Among the immunocompromised children, 15 (79%) received 3 doses of 10mcg BNT162b2 mRNA SARS-CoV-2 vaccine, while 4 (21%) received only 2 doses within 3 months of the first dose (**Figure 1A**). 1 child (chemotherapy group) dropped out of the study after receiving 2 doses of vaccine, and thus did not contribute data after the post-dose 1 time point. Otherwise, no subjects were excluded from analyses at any time point. Post-dose 2 data were combined from the 4 immunocompromised children who received a 2-dose primary vaccination regime, and 15 children who received a 3-dose primary regime, for both “post-dose 2” and “pre-dose 3” analyses. Several healthy and immunocompromised children went on to receive a 3rd and 4th dose, respectively, between month 6 and 12, according to parental discretion (**Figure 1A**).

### 3.2 Immunocompromised children have reduced immunogenicity to two vaccine doses, but this is improved upon after dose 3

We first compared the adaptive immune response to SARS-CoV-2 spike (S) protein after each of the first 2 doses of BNT162b2 between immunocompromised and healthy children. At each time point, children who acquired antigen rapid test positive (ART+) symptomatic SARS-CoV-2 infection, or asymptomatic SARS-CoV-2 (evidenced by serological or T cell responses), were excluded from analysis so that all comparisons were in children with vaccine-only immunity. After 2 doses, immunocompromised children had significantly lower anti-S IgG antibody titers and neutralizing antibodies titers against Wuhan-Hu-1 – measured by surrogate virus neutralization assay (sVNT) – compared to healthy children (**Figures 1B, C**). As we previously demonstrated that full protection against symptomatic BA.2 infection required a pVNT50 titer of 1000, we applied this titer as the protective pVNT50 threshold (1). We found no significant difference in pVNT50 titers against Beta and Delta variants, while pVNT50 titers were below the threshold of protection for all Omicron subvariants (**Figure 1D**).

To determine the impact of 2-dose vaccination on S+ MBCs and S-reactive T cell responses, we used the 80% sensitivity point on our receiver operating characteristic (ROC) curve that predicted protection against symptomatic SARS-CoV-2 infection (1). S+ MBCs were comparable after dose 2 in both cohorts (**Figure 1E**). However, S-reactive T cell responses (measured by interferon (IFN)γ using a previously reported cytokine release assay (1, 7), were significantly lower in immunocompromised children after 2 doses (**Figure 1F**). Unlike in healthy children where 60% achieved the threshold of protection, only 20% of immunocompromised children reached this threshold (**Figure 1F**). While age, weight and gender did not correlate with the measured immune responses, T cell response at day 10 post-dose 1 significantly correlated with total immunosuppression score (**Figure 1G**, **Supplementary Figures 1, 2**, **Supplementary Table 2**). This suggests that the reduced immunogenicity observed was more applicable to more severely immunosuppressed children.

Since a two-dose primary vaccination regime for immunocompromised children failed to produce S+ MBC and S-reactive T cells that correlated with protection, we analyzed the effect of the third dose by comparing the pre- to post-dose 3 parameters (**Figures 1H, J**). Expectedly, sVNT50 titers were significantly lower than that in healthy children (**Figure 1H**). As in healthy children, BA.2 pVNT50 titers remained below the protective threshold for the majority (67%) of immunocompromised children (**Figure 1I**). However, S-reactive T cell responses reached levels comparable to healthy children and importantly, above the threshold of protection (**Figure 1J**). Taken together, a third dose given as part of the primary vaccination regime to immunocompromised children in our cohort significantly improved vaccine immunogenicity.

### 3.3 Longevity of hybrid immunity is reduced in immunocompromised children compared to healthy children

Coincident with our study, community transmission of COVID-19 in Singapore fortuitously switched from low to high in December 2021, when Omicron became the predominant variant. In both healthy and immunocompromised children who received at least 1 dose of vaccine, proportion of asymptomatic infections was similar (26% in healthy, 30% in immunocompromised); symptomatic SARS-CoV-2 infections were all mild.

With endemicity of SARS-CoV-2, hybrid immunity will be the most frequent type of protection against COVID-19 globally. We thus compared hybrid immunity between immunocompromised and healthy children by using 12 immunocompromised children who had hybrid immunity at month 3 after 3 doses of vaccine and acquired one to two episodes of natural infection, and 54 healthy children who had hybrid immunity at month 3 after 2 doses of vaccine and acquired one to two episodes of natural infection. Children who acquired symptomatic/asymptomatic SARS-CoV-2 infection, or received booster doses of vaccine before each time point were excluded from analysis to allow us to assess the kinetics of hybrid immunity in the absence of further antigenic encounter.

**Figure 2:**
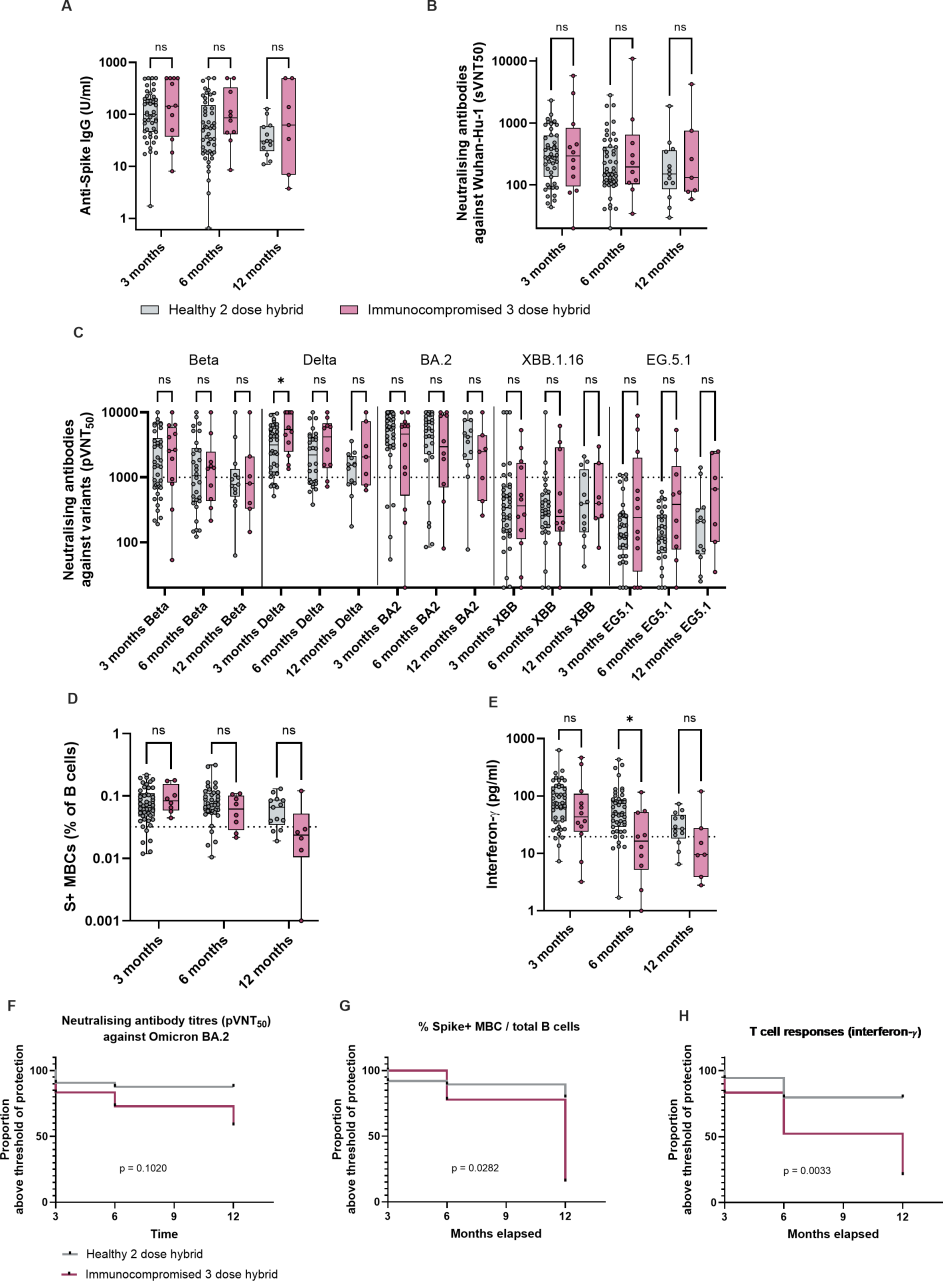
Longevity of hybrid immunity is reduced in immunocompromised children compared to healthy children. Immunological parameters of healthy and immunocompromised children with hybrid immunity, at month 3, 6 and 12 after completion of primary vaccination regime. 12 immunocompromised children had hybrid immunity at month 3 after having received 3 doses of vaccine and acquired one to two episodes of natural infection, and 54 healthy children. Children who acquired natural SARS-CoV-2 infection (both symptomatic and asymptomatic) or received a booster dose before month 6 and 12 were excluded from comparison for that time point. Thus, for month 6, n = 10 immunocompromised and n = 54 healthy, and for month 12, n = 7 immunocompromised and n = 17 healthy. **(A)** Anti-S IgG titers of healthy and immunocompromised children with hybrid immunity at month 3, with month 6 and month 12 titers for those who did not encounter further antigenic exposures. **(B)** sVNT_50_ against Wuhan-Hu-1. **(C)** pVNT_50_ against variants of concern. **(D)** S+ MBCs out of total B cells. **(E)** S-reactive T cell responses measured by post-stimulation IFN-γ. For all box-and-whisker graphs, the top and bottom boundaries of the boxes indicate upper and lower quartiles, respectively, the center line indicates median and the whiskers represent the range. For all panels, a two-tailed Mann-Whitney U test was used for comparisons between groups. Ns, not significant, *P ≤ 0.05. **(F)** Proportion of subjects who had Omicron BA.2 pVNT50 titers above the threshold of protection, between month 3, when hybrid immunity was first attained, and month 12. **(G)** Proportion of subjects who had S+ MBC responses above the threshold of protection. **(H)** Proportion of subjects who had S-reactive T-cell responses. Comparison of Kaplan Meier curves was analyzed with Logrank (Mantel-Cox) test.

Healthy and immunocompromised children demonstrated comparable anti-S IgG, neutralizing antibody titers, S+ MBCs and S-reactive T cell responses at month 3 (**Figures 2A–E**). However, by month 12, only 58% of immunocompromised children still had BA.2 neutralizing titers above the threshold of protection (**Figure 2F**). As we have previously shown that in the absence of protective titers of neutralizing antibodies, MBC and T cell responses are strongly predictive of protection against symptomatic infection, we analyzed the durability of these responses. Immunocompromised children demonstrated significantly more decline in S+ MBC and T cell responses than healthy children by month 12 (**Figures 2G, H**); the majority (84% for MBC and 79% for T cells) had MBC and T cell responses below the threshold of protection (**Figures 2G, H**). Taken together, we observed that, despite an initial robust formation of hybrid immun ity in immunocompromised children, the durability of immunological memory reduced significantly. This suggests that a booster dose given between 6 and 12 months after completion of primary vaccination regime may be beneficial in this population.

## 4 Discussion

Two previous studies in immunocompromised children which showed that the third dose of the primary vaccination regime elicited greater adaptive immune responses (5, 9) were conducted before correlates of protection against symptomatic SARS-CoV-2 infection were defined. Thus, the risk benefit ratio of a 3rd dose remained uncertain until now.

We found that a 3rd dose of BNT162bd did not augment neutralizing antibody titers, but elevated T cell responses above the protective threshold. As we have previously demonstrated T cell responses to be the most important predictor for clinical protection, especially in the context of antigenic mismatch between vaccine and circulating strains, this finding supports a 3-dose primary vaccination regime for immunocompromised children (1).

The majority of our cohort received T cell immunosuppressants (e.g., tacrolimus, azathioprine and methotrexate) throughout follow up. While this did not prevent the formation of robust hybrid immunity in all compartments of adaptive immunity, the longevity of hybrid immunity was reduced compared to healthy children. As the majority of the immunocompromised children had MBC and T cell responses below the threshold of protection by month 12, children whose neutralizing antibody titers fell below protective levels were at high risk of symptomatic SARS-CoV-2 infection. Thus, in contrast to healthy children, there may be a role for a booster dose in immunocompromised children 6 to 12 months after completion of primary vaccination.

Our study was limited by a small sample size of 19 children which was heterogeneous in terms of underlying disease etiology and immunosuppressants received. Too few received a booster dose, thus the immunological benefits of this could not be directly assessed. Additionally, pre-existing immunity due to prior exposure to seasonal coronaviruses was not considered in this study. Finally, we did not assess mucosal immunity, which is an important compartment of adaptive immunity in subjects who previously acquired natural SARS-CoV-2 infection. While this study focused on immunogenicity, future investigations assessing long-term clinical outcomes are necessary for a more comprehensive understanding of the protective effects of mRNA vaccines for immunocompromised children.

In conclusion, using previously defined correlates of protection against symptomatic SARS-CoV-2 infection, immunocompromised children may benefit from a 3-dose primary vaccination regime, with yearly or even twice-yearly booster doses for sustained immunity.

## Data Availability

The original contributions presented in the study are included in the article/**Supplementary Material**, further inquiries can be directed to the corresponding author/s.
