# Supplementary material for: Reduced durability of hybrid immunity to SARS-CoV-2 in immunocompromised children
Source: Front Immunol. 2024 Dec 17;15:1502598. doi: 10.3389/fimmu.2024.1502598 (PMC11685208; doi:10.3389/fimmu.2024.1502598)
Supplement: Supplementary file 1 [file DataSheet1.docx]

Reduced durability of hybrid immunity to SARS-CoV-2 in immunocompromised children

Youjia Zhong^1,2,3*^, Amuthavalli Kottaiswamy^1^, Chen Xiang Ang^3^, Hui’En Li^1^, Gaik Chin Yap^1^, Carina JX Tay^1^, Nurul Elyana Osman^1^, Siti Namirah Binte Roslan^1^, Chee Wah Tan^4^, Wee Chee Yap^4^, Elizabeth Y Ang^3^, Pauline PL Chan-Ng^1,3^, Hui Kim Yap^1,3^, Liangjian Lu^3^, Marion M Aw^1, 3^, Sivaraman V Karthik^3^, Seng Hock Quak^3^, Thuan Chong Quah^3^, Elizabeth H Tham^1,3^, Lynette P Shek^1,3#^, Eng Eong Ooi^2,5,6#*^

^#^These authors contributed equally

*** Correspondence:**Youjia Zhong
[youjiazhong@nus.edu.sg](mailto:youjiazhong@nus.edu.sg)

Eng Eong Ooi

[engeong.ooi@duke-nus.edu.sg](mailto:engeong.ooi@duke-nus.edu.sg)

Supplementary Material

# Supplementary Tables

## Supplementary Table 1. Participant Profile: Baseline demographic characteristics, vaccination status and immunosuppressive medications. Two-tailed T-test was used for comparisons between all continuous data and Fisher’s Exact Test was used for comparisons between all categorical data.

| **Characteristic** | **All Healthy Participants**  **(n = 116)** | **All Immuno-compromised Participants**  **(n = 19)** | ***P*-value** | **Post Solid Organ Transplantation**  **(n = 8)** | **Autoimmune Disease**  **(n = 8)** | **Acute Lymphoblastic Leukaemia**  **(n = 3)** |
| --- | --- | --- | --- | --- | --- | --- |
| Median age in years, (Range) | 8  (5 – 12) | 8  (5 – 12) | 0.59 | 8  (6 – 11) | 10  (7 – 12) | 5  (5 – 9) |
| Mean body weight in kilograms (Range) | 31.0  (16.0 – 68.1) | 25.6  (16.8 – 39.8) | < 0.05 | 24.5  (21.5 – 28.0) | 28.4  (20.0 – 39.8) | 20.9  (16.8 – 26.4) |
| Mean no. of days followed up (Range) | 483.5  (114.0 – 638.0) | 499.9  (21.0 – 673.0) | 0.61 | 557.9  (514.0 – 673) | 507.5  (425.0 – 657.0) | 325.0  (21.0 – 502.0) |
| Sex, n (%)  Male  Female | 46 (39.7)  70 (60.3) | 10 (52.6)  9 (47.4) | 0.41 | 3 (37.5)  5 (62.5) | 5 (62.5)  3 (37.5) | 2 (66.7)  1 (33.3) |
| Ethnicity, n (%)  Chinese  Indian  Malay  Others | 89 (76.6)  6 (5.2)  10 (8.6)  11 (9.5) | 9 (47.4)  3 (15.8)  4 (21.1)  3 (15.8) | 0.051 | 3 (37.5)  1 (12.5)  3 (37.5)  1 (12.5) | 6 (75.0)  1 (12.5)  0 (0.0)  1 (12.5) | 0 (0.0)  1 (33.3)  1 (33.3)  1 (33.3) |
| Vaccination Status, n (%)  2 vaccine doses  3 vaccine doses | 70 (60.3)  46 (39.7) | 4 (21.1)  15 (78.9) | < 0.05 | 0 (0.0)  8 (100.0) | 2 (25.0)  6 (75.0) | 2 (66.7)  1 (33.3) |
| Immunosuppressive medications, n (%)  Cyclosporin  Tacrolimus  Sirolimus  Azathioprine  Mycophenolate Mofetil  6-Mercaptopurine  Methotrexate  Prednisolone  IV Methylprednisolone*  IV Rituximab* | N.A. | 3 (15.8)  6 (31.6)  2 (10.5)  1 (5.3)  10 (52.6)  2 (10.5)  1 (5.3)  6 (31.6)  1 (5.3)  3 (15.8) | N.A. | 0 (0.0)  6 (31.6)  2 (10.5)  0 (0.0)  4 (21.1)  0 (0.0)  0 (0.0)  3 (15.8)  0 (0.0)  0 (0.0) | 3 (15.8)  0 (0.0)  0 (0.0)  1 (5.3)  6 (31.6)  0 (0.0)  1 (5.3)  3 (15.8)  1 (5.3)  3 (15.8) | 0 (0.0)  0 (0.0)  0 (0.0)  0 (0.0)  0 (0.0)  2 (10.5)  0 (0.0)  0 (0.0)  0 (0.0)  0 (0.0) |

***Footnotes:***

*Solid organ transplantation: n = 7 liver transplant, n = 1 renal transplant, all on immunosuppression at recruitment*

*Autoimmune disease: n = 5 Nephrotic Syndrome, n = 1 Crohn’s Disease, n = 1 Systemic Lupus Erythematosus, n = 1 Juvenile Polymyositis, all on immunosuppression at recruitment*

*Acute lymphoblastic leukaemia: n = 1 ongoing chemotherapy, n = 2 completed treatment within last 3 months*

*All immunosuppressive medications that the patient was regularly taking 1 month before first dose to 3 months after last dose of vaccine were included*

**: this medication was given as a short course, or single dose, 1 month before first dose and 3 months after last dose of vaccine*

**Supplementary Figure 1**: Immunological parameters in immunocompromised children analyzed in association to demographic features after dose 1.Scatterplots (A–K) display the correlation coefficients and p-values for immunological parameters such as Anti-S IgG, S+ MBCs, S-reactive T cell responses in immunocompromised children with vaccine-only immunity, analyzed against demographic features such as age, weight, gender, and total immunosuppression score after Dose 1. Spearman correlation and two-tailed Mann-Whitney U tests were used for statistical evaluation.

**Supplementary Figure 2**: Immunological parameters in immunocompromised children analyzed in association to demographic features after dose 2.Scatterplots (A–L) show the correlation coefficients and p-values for immunological parameters such as Anti-S IgG, sVNT_50_ against Wuhan-Hu-1, S+ MBCs and S-reactive T cell responses in immunocompromised children with vaccine-only immunity, analyzed against demographic features such as age, weight, and total immunosuppression score after Dose 2. Spearman correlation was used to assess statistical significance. Gender based correlation was not applicable after 2 dose group because the sample size was not sufficient for two-tailed Mann-Whitney U test.

| **Immunosuppressant** | **Vasudev Score** | **Höcker Score** | **MARVELS total immunosuppression score** |
| --- | --- | --- | --- |
|  | **Dose per unit (mg/day)** | **Dose per unit**  **(mg/m^2^/day)** | **Dose per unit (mg/m^2^/day)** |
| Tacrolimus | 2 | 1.2 | 1.2 |
| Cyclosporin microemulsion | 100 | 58 | 59 |
| Sirolimus | 2 | 1.2 | 1.2 |
| Mycophenolate mofetil | 500 | 290 | 291 |
| Azathioprine | 100 | 58 | 59 |
| Prednisone equivalent | 5 | 2.9 | 2.9 |
| Methylprednisolone | 4 | 2.3 | 2.3 |
| Dexamethasone | 0.8 | 0.45 | 0.45 |
| Methotrexate | - | - | 3 |
| 6-Mercaptopurine | - | - | 6 |

## Supplementary Table 2. Total immunosuppression score for immunosuppressive medications used in the MARVELS immunocompromised cohort, based on Vasudev et al (2005) and Höcker et al (2012).
