# Supplementary material for: Reduced durability of hybrid immunity to SARS-CoV-2 in immunocompromised children
Source: Front Immunol. 2024 Dec 17;15:1502598. doi: 10.3389/fimmu.2024.1502598 (PMC11685208; doi:10.3389/fimmu.2024.1502598)
Supplement: Supplementary file 2 [file DataSheet2.docx]

# A B C

r= -0.154

**100**

r= -0.278

**1**

**p=0.313**

**p=0.485**

Spike+ MBCs (% of B cells)

r= -0.223

**1000**

**100**

**p=0.701**

**S-reactive T cell response (IFN**ψ**) [pg/ml])**

**0.1**

Anti-Spike IgG (U/ml)

**10 10**

**1**

**0 5 10 15**

Age

**0.01**

**0.001**

**0 5 10 15**

Age

**1**

**0.1**

**0 5 10 15**

Age

# D E F

**100**

Anti-Spike IgG (U/ml)

**10**

**1**

r= -0.348

**p=0.202**

**0 10 20 30 40 50**

Weight

**1**

**0.1**

Spike+ MBCs (% of B cells)

**0.01**

**0.001**

r= -0.356


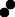


**p=0.256**

**0 10 20 30 40 50**

Weight

**1000**

**100**

S-reactive T cell response (IFNψ) [pg/ml])

**10**

**1**

**0.1**

r= -0.490


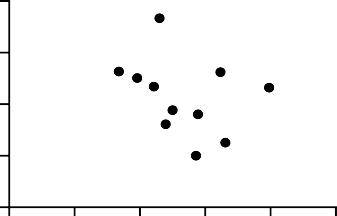


**p=0.129**

**0 10 20 30 40 50**

Weight

# G H I

**Spike+ MBCs (% of B cells)**

**100**

## p=0.284 1

## p=0.937

**1000**

## p=0.792

**10**

**Anti-Spike IgG (U/ml)**

**1**

**Male Female**

**0.1**

**0.01**

**0.001**

**Male Female**

**100**

**10**

**1**

**0.1**

**Male Female**

**S-reactive T cell response (IFN**ψ**) [pg/ml])**

# J K

**100**

r= -0.504

**1**

**p=0.056**


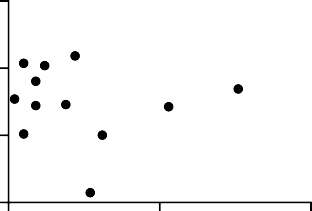


**p=0.438**

**Spike+ MBCs (% of B cells)**

**r= -0.245**

**0.1**

Anti-Spike IgG (U/ml)

**10**

**0.01**

**1**

**0 5 10**

Total immunosuppression score

**0.001**

**0 5 10**

**Total immunosuppression score**
