# Supplementary material for: Reduced durability of hybrid immunity to SARS-CoV-2 in immunocompromised children
Source: Front Immunol. 2024 Dec 17;15:1502598. doi: 10.3389/fimmu.2024.1502598 (PMC11685208; doi:10.3389/fimmu.2024.1502598)
Supplement: Supplementary file 3 [file DataSheet3.docx]

# A

**100**

**p=0.350**

r= -0.600

**B**

**1000**

Neutralising antibodies against Wuhan-Hu-1 (sVNT50)

**C**

r= -0.205

p=0.733 1

% Classical S+ MBCs / total B cells

r= -0.200 p=0.783

**D**

**100**

S-reactive T cell response (IFNψ) [pg/ml])

r= -0.100 p=0.950

**0.1 10**

Anti-Spike IgG (U/ml)

**10 100**

**0.01 1**

**1**

**0 5 10 15**

Age

**10**

**5 10 15**

Age

**0.001**

**0 5 10 15**

Age

**0.1**

**5 10 15**

Age

# E

**100**

**p=0.950**

r= -0.100

**F**

**1000**

**p=0.900**

Neutralising antibodies against Wuhan-Hu-1 (sVNT50)

**G**

r= -0.102 1

% Classical S+ MBCs / total B cells

r= -0.700 p=0.233

**H**

**100**

S-reactive T cell response (IFNψ) [pg/ml])

r= -0.500 p=0.450

**10 100**

Anti-Spike IgG (U/ml)

**0.1 10**

**0.01 1**

**1**

**10 20 30 40**

Weight

**10**

**10 20 30 40**

Weight

**0.001**

**10 20 30 40**

Weight

**0.1**

**10 20 30 40**

Weight

# I

**100**

**p=1.00**

r= -0.000

**J**

**1000**

Neutralising antibodies against Wuhan-Hu-1 (sVNT50)

**K**

r= -0.359 1

**p=0.566**

% Classical S+ MBCs / total B cells

r= -0.500 p=0.450

**L**

**100**

S-reactive T cell response (IFNψ) [pg/ml])

r= -0.300 p=0.683

**10**

Anti-Spike IgG (U/ml)

**100**

**0.1 10**

**0.01 1**

**1**

**0 5 10 15**

**10**

**0 5 10 15**

**0.001**

**0 5 10 15**

**0.1**

**0 5 10 15**

Total immunosuppression score

Total immunosuppression score

Total immunosuppression score

Total immunosuppression score
